# IGF-1R inhibition induces schedule-dependent sensitization of human melanoma to temozolomide

**DOI:** 10.18632/oncotarget.5631

**Published:** 2015-10-15

**Authors:** Roger Ramcharan, Tamara Aleksic, Wilfride Petnga Kamdoum, Shan Gao, Sophia X. Pfister, Jordan Tanner, Esther Bridges, Ruth Asher, Amanda J. Watson, Geoffrey P. Margison, Mick Woodcock, Emmanouela Repapi, Ji-Liang Li, Mark R. Middleton, Valentine M. Macaulay

**Affiliations:** ^1^ Department of Oncology, Old Road Campus Research Building, Oxford, UK; ^2^ Biomedical Services, John Radcliffe Hospital, Oxford, UK; ^3^ Department of Pathology, John Radcliffe Hospital, Oxford, UK; ^4^ Cancer Research UK Carcinogenesis Group, Paterson Institute for Cancer Research, Manchester, UK; ^5^ Computational Biology Research Group, Weatherall Institute of Molecular Medicine, Oxford, UK; ^6^ Oxford Cancer Centre, Churchill Hospital, Oxford, UK

**Keywords:** IGF-1R, chemo-sensitization, double strand break, temozolomide, apoptosis

## Abstract

Prior studies implicate type 1 IGF receptor (IGF-1R) in mediating chemo-resistance. Here, we investigated whether IGF-1R influences response to temozolomide (TMZ), which generates DNA adducts that are removed by *O*^6^-methylguanine-DNA methyltransferase (MGMT), or persist causing replication-associated double-strand breaks (DSBs). Initial assessment in 10 melanoma cell lines revealed that TMZ resistance correlated with MGMT expression (*r* = 0.79, *p* = 0.009), and in MGMT-proficient cell lines, with phospho-IGF-1R (*r* = 0.81, *p* = 0.038), suggesting that TMZ resistance associates with IGF-1R activation. Next, effects of IGF-1R inhibitors (IGF-1Ri) AZ3801 and linsitinib (OSI-906) were tested on TMZ-sensitivity, cell cycle progression and DSB induction. IGF-1Ri sensitized BRAF wild-type and mutant melanoma cells to TMZ *in vitro*, an effect that was independent of MGMT. Cells harboring wild-type p53 were more sensitive to IGF-1Ri, and showed schedule-dependent chemo-sensitization that was most effective when IGF-1Ri followed TMZ. This sequence sensitized to clinically-achievable TMZ concentrations and enhanced TMZ-induced apoptosis. Simultaneous or prior IGF-1Ri caused less effective chemo-sensitization, associated with increased G1 population and reduced accumulation of TMZ-induced DSBs. Clinically relevant sequential (TMZ → IGF-1Ri) treatment was tested in mice bearing A375M (V600E BRAF, wild-type p53) melanoma xenografts, achieving peak plasma/tumor IGF-1Ri levels comparable to clinical Cmax, and inducing extensive intratumoral apoptosis. TMZ or IGF-1Ri caused minor inhibition of tumor growth (gradient reduction 13%, 25% respectively), while combination treatment caused supra-additive growth delay (72%) that was significantly different from control (*p* < 0.01), TMZ (*p* < 0.01) and IGF-1Ri (*p* < 0.05) groups. These data highlight the importance of scheduling when combining IGF-1Ri and other targeted agents with drugs that induce replication-associated DNA damage.

## INTRODUCTION

Metastatic melanoma is highly chemo-resistant: dacarbazine or temozolomide (TMZ) induce responses in <20% of patients, with very limited survival benefit [1, 2]. Approximately 40–50% of melanomas harbour activating mutations in the RAS-RAF axis, commonly V600E BRAF [3]. The outlook has improved significantly following development of novel forms of immunotherapy and mutant BRAF inhibitors, although responses to the latter are often brief, with early emergence of resistance [4, 5].

Type 1 insulin-like growth factor receptor (IGF-1R) is frequently up-regulated in cancers including melanoma, and promotes proliferation and cell survival [6, 7]. IGF-1R expression has been associated with an aggressive clinical course and resistance to chemotherapy and targeted agents [8–10]. We previously showed that IGF-1R depletion blocks survival of BRAF mutant and wild-type (WT) melanoma cells, and enhances chemosensitivity [11]. Recently, we reported that IGF-1R depletion or inhibition delay repair of radiation-induced DNA double-strand breaks (DSBs), with evidence of impaired repair by both non-homologous end-joining and homologous recombination (HR) [12, 13]. Many cytotoxic drugs cause DNA damage that is induced during DNA replication, requiring HR for repair [14]. Initiation of HR is very tightly coupled to cell cycle regulation, itself known to be influenced by IGFs [7, 15]. Therefore, as a tool to probe the relationship between IGF-1R and induction of replication-associated DNA damage, we examined responses to TMZ, a methylating agent used to treat glioblastoma multiforme and melanoma [16].

We tested two ATP-competitive IGF-1R tyrosine kinase inhibitors (IGF-1Ri): preclinical compound AZ12253801 (AZ3801), used in our study of effects of IGF-1Ri on the DNA damage response [13], and OSI-906 (linsitinib), developed for clinical evaluation [17–19]. AZ3801 has ~10 fold selectivity over the insulin receptor (INSR), with IC_50_ values for inhibition of IGF-1R of 2.1 nM and INSR 19 nM [13]. OSI-906 is ~2-fold more potent against IGF-1R than INSR (IC_50_ values 35 nM and 75 nM respectively), and recently completed Phase I trials using intermittent or continuous schedules [17–19]. Our data show an association between WT p53 status and sensitivity to IGF-1Ri, reveal that IGF-1R activation correlates with resistance to TMZ, and show that significant enhancement of chemo-sensitivity is induced by a sequential (TMZ → IGF-1Ri) schedule. In contrast, IGF-1Ri pre-treatment causes less effective chemo-sensitization, and we show for the first time that pre-inhibiting IGF-1R induces cell cycle delay associated with reduced yield of toxic chemotherapy-induced DNA damage. These findings have clear relevance to the design of future trials of IGF axis inhibitors with chemotherapy.

## RESULTS

### IGF-1R phosphorylation associates with resistance of melanoma cell lines to TMZ

**Figure 1:**
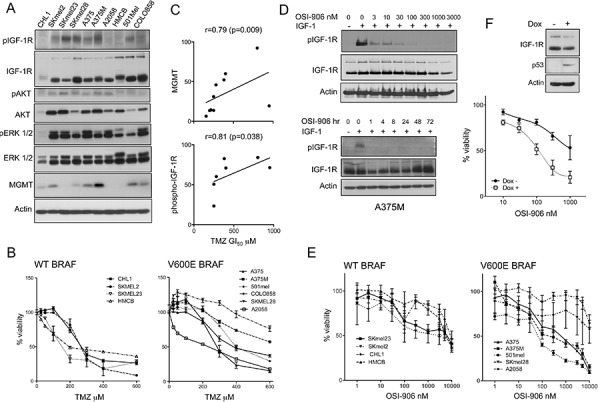
IGF axis association with TMZ resistance and p53 status **A.** Whole cell extracts analyzed by western blotting. Similar results were obtained in a second set of independent lysates. **B.** Cells were treated with TMZ or vehicle, after 5 days CellTiter Glo viability assays were performed and data were expressed as % viability in TMZ-untreated cultures. Graphs: pooled results from 2 independent assays (6 data points) expressed as mean ± SEM % viability. **C.** Correlation between TMZ GI_50_ and upper: MGMT; lower: phospho-IGF-1R, quantified from western blots in A, corrected for actin loading. **D.** Serum-starved A375M cells treated with: upper, OSI-906 for 1 hr; lower: 1 μM OSI-906 for 1–72 hr, and in the final 15 min with 50 nM IGF-1. **E.** Cells were treated with OSI-906 or vehicle, and after 5 days viability assayed as B). **F.** B231 cells treated with solvent or 2 μg/ml doxycycline for 24 hr, and lysed for western blot (inset) or treated with solvent or OSI-906 as E) and viability assayed after 5 days. Graph: data from *n* = 3 assays; bars, SEM. GI_50_ values were > 1 μM in p53 null cells, 116 nM in p53-positive cells.

In a human melanoma cell line panel we assessed levels and activation of IGF-1R and its effectors AKT and ERKs, and expression of *O*^6^-methylguanine-DNA methyltransferase (MGMT) that removes the most toxic TMZ-induced DNA adduct, *O*^6^-methyguanine (*O*^6^-meG; [16]; Figure 1A). All cell lines expressed IGF-1R, with variable receptor phosphorylation. Reflecting the frequency of RAS-RAF activation [3], phospho-ERK was detectable in all cell lines except CHL1 that harbors WT BRAF/NRAS (Table 1). In a first approach to test the relationship between IGF-1R and chemo-resistance, we evaluated TMZ in viability assays (Figure 1B, Table 1). All cell lines were relatively TMZ-resistant, with GI_50_ values 155–950 μM, above 60 μM, the clinically-achievable Cmax [1]. MGMT-deficient SKmel23, A2058 and HMCB were relatively sensitive (TMZ GI_50_ 155 - 215 μM), and MGMT-positive cell lines more resistant (TMZ GI_50_ ≥ 250 μM; Table 1). TMZ GI_50_ values significantly correlated with MGMT expression (*r* = 0.79, *p* = 0.009; Figure 1C, upper panel), and showed borderline correlation with IGF-1R protein levels (*r* = 0.64, *p* = 0.052, Supplementary Figure S1A). In 7 MGMT proficient cell lines, there was significant correlation between TMZ GI_50_ and phospho-IGF-1R (*r* = 0.81, *p* = 0.038; Figure 1C lower), suggesting association between activated IGF-1R and intrinsic TMZ resistance. These results prompted us to test effects of IGF-1Ri on growth and chemo-resistance.

**Table 1:** Characteristics of melanoma cell line panel

Cell line	BRAF	NRAS	PTEN	p53	Other	GI_50_	
						TMZ μM	OSI-906 nM
CHL1	WT	WT	WT	Mut	CDKN2A	250	9200
SKmel2	WT	Mut	WT	Mut	–	250	3420
SKmel23	WT	WT	Del	WT	–	155	5960
SKmel28	Mut	WT	Mut	Mut	EGFR	950	>10000
A375	Mut	WT	WT	WT	CDKN2A	280	800
A375M	Mut	WT	WT	WT	CDKN2A	800	150
A2058	Mut	WT	Mut	Mut	CDKN2A	200	>10000
HMCB	WT	Mut	WT	Mut	–	215	5880
501mel	Mut	WT	WT	Mut	CTNNB1	400	60
COLO858	Mut	WT	WT	WT	–	380	ND

Genotypes from http://www.sanger.ac.uk/genetics/CGP/CellLines/, www-p53.iarc.fr/CellLineCriteria.asp and references [30, 51, 52]. Data from cell viability assays (Figure 1B, 1E) were curve-fitted to interpolate TMZ and OSI-906 GI_50_ values.

### Cells that harbor WT p53 are more sensitive to IGF-1R inhibition

Initial experiments tested sensitivity to IGF-1R inhibitor OSI-906, which was shown to be capable of blocking IGF-1R activation for ≥72 hr in A375M cells (Figure 1D). In the cell line panel, OSI-906 caused variable concentration-dependent inhibition of melanoma cell viability (Figure 1E), with GI_50_ values from the nanomolar to low micromolar range (Table 1). These are clinically achievable concentrations: continuous OSI-906 dosing at 150 mg BID achieves plasma levels of ~1000–2000 ng/ml (2.4–4.8 μM), while 600 mg OSI-906 intermittently achieves Cmax of ~8000 ng/ml (~20 μM), remaining at 24 hr above 1 μM, predicted to be required for efficacy [18, 19]. Here, there was no correlation between OSI-906 sensitivity and total/activated IGF-1R in the melanoma cell lines, and no evidence that downstream pathway activation due to PTEN loss, BRAF or NRAS mutation was associated with IGF-1Ri resistance, consistent with our previous data using *IGF1R* gene silencing [11]. We noted that the three most IGF-1Ri-resistant cell lines (GI_50_ ~10 μM or greater) harbored mutant p53 (Table 1). Given that these cell lines are genetically heterogeneous, we tested the relationship between p53 and response to IGF-1Ri in p53-null B231 cells, in which expression of WT p53 was induced by doxycycline (Figure 1F; [20]. WT p53 is reported to suppress IGF-1R expression [21], but did not influence IGF-1R levels here. The p53 null cells were relatively resistant to OSI-906 (GI_50_ > 1 μM), and doxycycline-treated cells more sensitive (GI_50_ 116 nM), indicating ≥8-fold sensitization to OSI-906. Thus in both this isogenic model and the melanoma cell lines, lack of WT p53 correlated with relative resistance to IGF-1Ri.

### IGF-1R inhibition induces MGMT-independent sensitization of BRAF WT and mutant melanoma cells to TMZ

**Figure 2:**
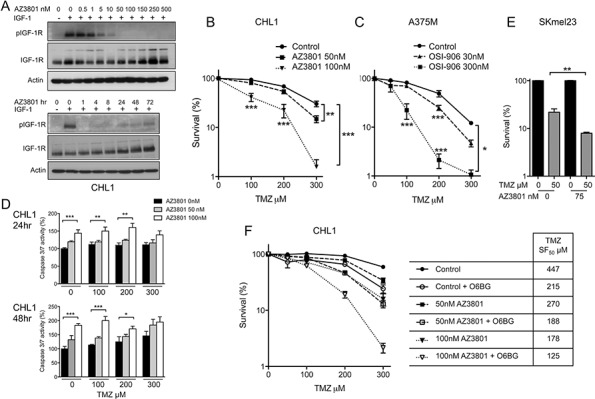
IGF-1R inhibition induces MGMT-independent sensitization of BRAF WT and mutant melanoma cells to TMZ **A.** Serum-starved CHL1 cells treated with: upper, AZ3801 for 1 hr; lower, 30 nM AZ12253801 for 1–72 hr, and with IGF-1 as Figure 1D. **B.** CHL1 and **C.** A375M cells treated with TMZ alone or with AZ3801 or OSI-906. Graphs: mean ± SEM % cell survival from *n* = 3 independent assays in each cell line. IGF-1Ri-treated cells were sensitized to TMZ (*p < 0.05, **p < 0.01, ***p < 0.001). **D.** Graphs: mean ± SEM caspase activity (% signal in solvent-treated controls) from *n* = 2 independent assays in CHL1 cells, each with triplicate samples. AZ3801 enhanced apoptosis after 24 hr (upper) and 48 hr (lower; *p < 0.05, **p < 0.01, ***p < 0.001). **E.** SKmel23 cells treated with solvent or 50 μM TMZ alone or with 75 nM AZ3801. Graph: mean ± SEM % survival from *n* = 3 assays (*p < 0.05). The sensitivity of SKmel23 to IGF-1Ri precluded testing of AZ3801 > 75 nM. **F.** CHL1 cells pre-treated for 2 hr with 10 μM O6BG prior to TMZ alone or with AZ3801. Graph: pooled data from *n* = 2 survival assays from which were derived TMZ SF_50_ values, shown in legend. In cultures treated with 300 μM TMZ, O6BG suppressed survival of AZ3801-untreated controls from 59 ± 3% to 24 ± 4% (*p* < 0.001), with 50 nM AZ3801 from 34 ± 4% to 13 ± 2% ( *p* < 0.001), and 100 nM AZ3801 from 16 ± 4% to 2 ± 0.5% ( *p* < 0.05).

We next assessed whether IGF-1Ri modifies response to TMZ *in vitro*, testing OSI-906 and AZ3801 in BRAF mutant A375M and BRAF WT CHL1, relatively TMZ-resistant (TMZ GI_50_ 800 μM) and sensitive (250 μM), respectively (Table 1). Like OSI-906, AZ3801 caused dose-dependent IGF-1R inhibition for up to 72 hr (Figure 2A). On testing responses to TMZ alone or with IGF-1Ri in survival assays, both OSI-906 and AZ3801 induced dose-dependent TMZ sensitization in each cell line (Figure 2B, 2C, Supplementary Figure 1B–1C). The effect size was similar with both inhibitors, and comparable to TMZ-sensitization we previously reported in IGF-1R-depleted melanoma cells [11], supporting the contention that sensitization was related to IGF-1R inhibition. To explore the molecular basis for chemosensitization, we performed caspase 3/7 activity assays (Figure 2D) and western blotting for PARP cleavage (Supplementary Figure S2A), detecting increased TMZ-induced apoptosis in IGF-1R inhibited cells after 24–48 hr. However, the effect of AZ3801 on apoptosis did not increase with TMZ concentration, unlike the pattern of chemo-sensitization (Figure 2B–2C), suggesting that chemo-sensitization was unlikely to be wholly attributable to apoptosis induction at these early time-points. Therefore, we next investigated whether IGF-1R influences repair of TMZ-induced DNA damage.

The most toxic alkylated base lesion produced by TMZ is *O*^6^-meG, which can be directly repaired by methyl group transfer to a cysteine residue in the MGMT active site pocket [16]. MGMT downregulation is associated, as here (Figure 1C upper), with melanoma sensitivity to TMZ *in vitro*, although not clinically [22]. Other base lesions include N7-meG, the commonest TMZ-induced lesion, which undergoes spontaneous depurination to generate toxic apurinic (AP) sites, and N3meA, which is intrinsically cytotoxic. Both lesions can be excised by 3-alkyladeinine DNA glycosylase (AAG) [23]. To test whether IGF-1Ri influences MGMT and AAG, we first performed chemo-sensitivity assays in MGMT-null SKmel23 cells, which are highly TMZ sensitive, but even so IGF-1R induced further sensitization (Figure 2E). Secondly, in MGMT-proficient CHL1 and A375M, MGMT activity/expression was unaffected by IGF-1Ri (Supplementary Figure 2B left panel, 2C). MGMT assay specificity was supported by abolition of MGMT activity in CHL1 and A375M by MGMT substrate analogue *O*^6^-Benzylguanine (O6BG), and by undetectable MGMT activity in SKmel23 (Supplementary Figure 2B left panel). Similarly, IGF-1Ri did not influence AAG activity or TMZ-induced *O*^6^-meG or N7-meG adducts (Supplementary Figure 2B right panel, 2D–2E). Furthermore, AZ3801 was capable of sensitizing to TMZ in CHL1 cells in which MGMT was inhibited by O6BG (Figure 2F). These results suggest that with respect to TMZ-sensitization, IGF-1R does not function in the same pathway as MGMT.

### IGF-1R inhibition induces schedule-dependent chemo-sensitization of melanoma cells

**Figure 3:**
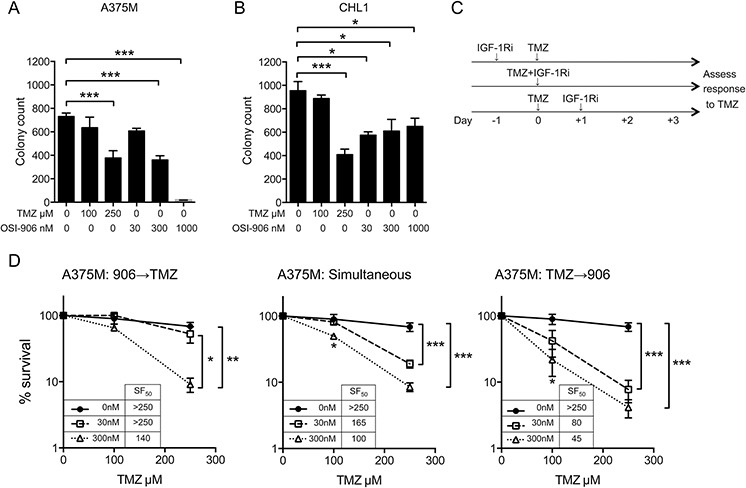
IGF-1R inhibition induces schedule-dependent sensitization to TMZ **A.** A375M, **B.** CHL1 cells were treated with solvent, OSI-906 or TMZ. Graphs: mean ± SEM % survival from *n* = 2 assays, each with triplicate dishes (6 data points). Cell survival was inhibited in both cell lines by 250 μM TMZ and by OSI-906 at 300–1000 nM in A375M, and 30–1000 nM in CHL1 (*p < 0.05, ***p < 0.001). **C.** Treatment schedules for chemo-sensitivity testing. **D.** A375M cells treated simultaneously or sequentially with solvent, 30–300 nM OSI-906 and/or TMZ. Graphs: mean ± SEM % survival from *n* = 3 assays, showing significance of differences from control (OSI-906-untreated) cultures (*p < 0.05, **p < 0.01, ***p < 0.001). Legends: TMZ SF_50_ (μM) at each OSI-906 concentration.

If MGMT levels are inadequate, *O*^6^-meG persists and undergoes post-replicative mispairing to form G:T pairs that are substrates for mismatch repair. Repeated ‘futile cycles’ of exonuclease activity and mispairing lead to extension of single-stranded gaps, resulting after ≥2 rounds of replication in replication fork collapse and formation of DSBs [16]. Given that IGFs are well-known to promote cell cycle progression [7], it is plausible that inhibiting this function could influence accumulation of replication-associated DSBs. As an initial step to investigate interactions between the IGF axis and TMZ-response, we tested whether chemo-sensitization is influenced by sequencing IGF-1Ri with respect to TMZ. Based on initial chemo-sensitivity data (Figure 2B–2C), we first tested each drug separately to choose concentrations for combination experiments, using TMZ at 100–250 μM, and OSI-906 at 300–1000 nM in CHL1 and 30–300 nM in A375M; 1000 nM was too toxic in these cells for combination testing (Figure 3A–3B). We next tested whether chemo-sensitization is influenced by sequencing of IGF-1Ri, using schedules illustrated in Figure 3C. There was no evidence of antagonism (ie no increase in TMZ SF_50_ in OSI-906-treated cells), although cells pre-treated with 30 nM OSI-906 were no more TMZ-sensitive than controls. With this exception, IGF-1R-inhibited cells showed dose-dependent sensitization to TMZ (Figure 3D), most effectively when OSI-906 was applied 24 hr after TMZ, compared with cells that were pre-treated or simultaneously-treated. Notably, post-treatment with 300 nM OSI-906 achieved TMZ SF_50_ of 45 μM (Figure 3D, right panel), representing > 5.5 fold sensitization compared with control (OSI-906-untreated) cells. This SF_50_ value is below the Cmax of TMZ (~11 μg/ml, ~60 μM) administered clinically as a single agent [1]. Equivalent experiments in CHL1 cells confirmed TMZ-sensitization, but there was no difference in efficacy when IGF-1Ri was added before, concurrently or after TMZ (Supplementary Figure 3A–3B).

### IGF-1R inhibition influences cell cycle distribution and DNA damage induction post-TMZ

**Figure 4:**
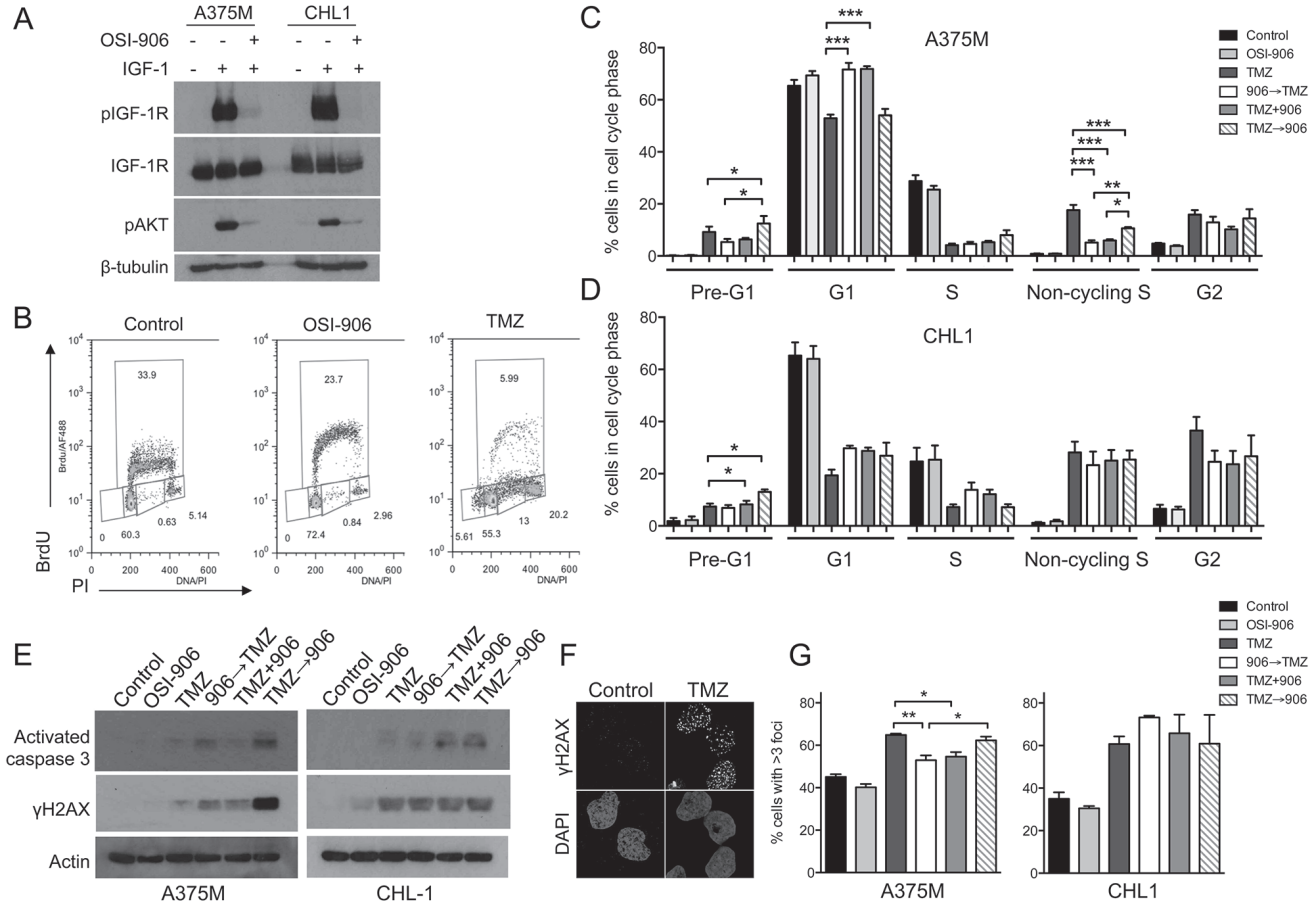
IGF-1R inhibition pre-TMZ induces G1 arrest and reduces DSB induction **A.** Cells were treated with 300 nM OSI-906 and IGF-1 as Figure 1D. **B.** A375M cells treated with solvent, 300 nM OSI-906 or 300 μM TMZ. After 72 hr, cell cycle profiles were determined by analysis of BrdU incorporation vs DNA content (PI staining). **C.** A375M, **D.** CHL1 cells treated with solvent, 300 nM OSI-906, 300 μM TMZ alone or with 300 nM OSI-906 applied 24 hr before, simultaneously or 24 hr post-TMZ, and collected 72 hr post-TMZ. Graphs: mean ± SEM data from *n* = 4 analyses in each cell line. Compared with TMZ alone, TMZ-treated/IGF-1R-inhibited cells showed significant differences in pre-G1, G1 and non-cycling S populations of A375M, and only in the pre-G1 population of CHL1 (*p < 0.05, **p < 0.01, ***p < 0.001). **E.** Cultures treated as C, D were lysed 72 hr post-TMZ for western blotting. **F.** CHL1 cells were treated with solvent or 300 μM TMZ and after 72 hr stained for γH2AX. **G.** Cells were treated as C), D) and after 120 hr, 50–2300 cells per condition were analysed for γH2AX foci. Graphs: mean ± SEM % cells with > 3 foci. Pre- or co- inhibition of IGF-1R significantly reduced TMZ-induced foci in A375M (*p < 0.05, **p < 0.01 compared with TMZ alone).

Aiming to understand the basis for schedule-dependent chemo-sensitization of A375M cells and the lack of sequence variation in CHL1, we investigated changes in cell cycle distribution. After 24 hr exposure to OSI-906 or AZ3801, there was increase in G1 and reduction in S phase fraction (Supplementary Figure S4A–S4B), consistent with the ability of IGFs to promote G1 to S transition [7]. TMZ-treated cells showed an increased G2 population and progressive reduction in S-phase, also evident upon combination treatment and most pronounced at 72 hr (Supplementary Figure S4A–S4B). Fixing on this 72 hr post-TMZ time-point, we tested effects of altering the sequence of IGF-1Ri with respect to TMZ, using the same schedule (Figure 3C) as for chemo-sensitivity testing. These experiments used 300 nM OSI-906, confirmed to inhibit IGF signaling in both cell lines (Figure 4A). In A375M, TMZ-induced reduction in S-phase and increase in G2 was unaffected by IGF-1R pre-, co- or post- inhibition (Figure 4B–4C). However, the G1 population was significantly increased when IGF-1R was pre- or co- inhibited, compared with TMZ alone and with IGF-1R post-inhibition (Figure 4C). TMZ induced appearance of non-cycling S-phase cells with DNA content between 2N and 4N but without BrdU incorporation (Figure 4C), suggesting intra-S checkpoint activation [24]. This population was reduced by IGF-1Ri; further work is required to test whether this represents loss of checkpoint integrity. We noted appearance of pre-G1 (likely apoptotic) DNA content, significantly greater in IGF-1R post-inhibited cells, correlating with the pattern of chemo-sensitization (Figure 3D). In contrast, IGF-1Ri-treated CHL1 cells showed no change in cell cycle distribution of viable cells, although there was an increase in the pre-G1 population in IGF-1R post-inhibited cells compared with TMZ alone (Figure 4D). To check whether this represented increased apoptosis, we assessed caspase activation by western blot (Figure 4E). TMZ alone generated little positive signal in either cell line, with an increase on addition of OSI-906, particularly upon post-treatment in A375M. We also probed for DNA damage marker γH2AX, noting that relative γH2AX signals in OSI-906 pre, co- and post- treated A375M cells (Figure 4E left) paralleled the pattern of relative chemo-sensitization (Figure 3D), with more intense signal upon IGF-1R post-inhibition. In CHL1, where sequencing did not influence chemo-sensitivity, IGF-1Ri did not influence TMZ-induced γH2AX (Figure 4E right).

The γH2AX western blot suggested possible excess DNA damage in A375M cells treated with OSI-906 after TMZ. Although principally induced by DSBs, non-focal γH2AX can be induced by other processes including apoptosis [25, 26]. Therefore, we assessed immunofluorescent γH2AX foci formed at sites of DSBs. Testing 72 hr post-TMZ to parallel cell cycle and western blotting data, we detected γH2AX foci in TMZ-treated cells (Figure 4F), and quantified changes in cells exposed to TMZ alone or with pre-, co- or post- treatment with OSI-906, as Figure 3C. As a single agent, TMZ induced more foci in CHL1 than A375M cells (Supplementary Figure S4C), consistent with relative TMZ sensitivities in viability assays (Table 1). There was no difference in TMZ-induced foci in IGF-1Ri-treated A375M or CHL1 cells at this 72 hr time-point (Supplementary Figure S4C). In light of the cell cycle changes seen previously (Figure 4C), and the time needed to generate toxic DSBs, we repeated this assessment 120 hr post-TMZ. Here, we observed significant reduction in TMZ-induced γH2AX foci in A375M cells pre- or co-treated with OSI-906 compared with cells treated with TMZ alone or TMZ followed by OSI-906 (Figure 4G, left). These findings suggest reduced induction of DNA damage as a consequence of G1 accumulation of IGF-1R pre- or co- inhibited cells. In contrast, IGF-1R inhibition did not influence TMZ-induced γH2AX foci in CHL1 cells (Figure 4G, right), in which there were no sequence-dependent changes in TMZ-sensitization (Supplementary Figure S3) or cell cycle distribution (Figure 4D).

### OSI-906 enhances sensitivity of melanoma xenografts to temozolomide

**Figure 5:**
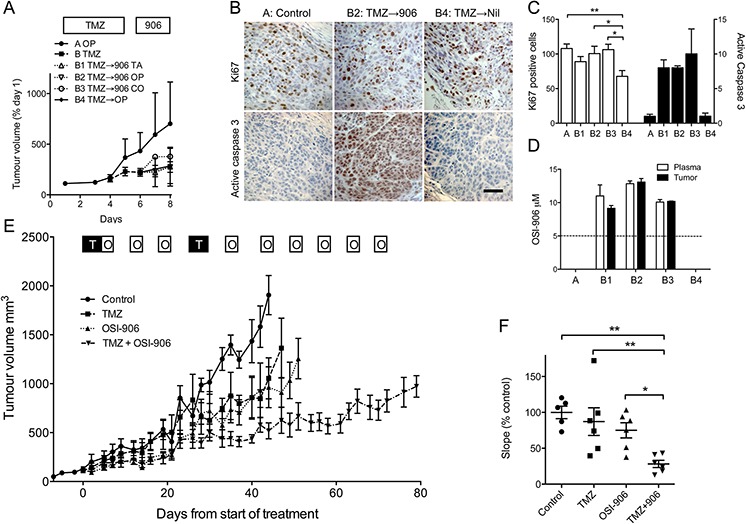
IGF-1R inhibition sensitizes melanoma to TMZ *in vivo* **A.** Mice bearing A375M xenografts were treated with Ora-Plus (OP, Group A, 2 mice) or 50 mg/kg TMZ in Ora-Plus by gavage days 1–5 (Group B, 8 mice). On day 6, group B was randomly divided into 4 groups of 2 for gavage on days 6–8 with 50 mg/kg OSI-906 in 25 mM tartaric acid (TA, B1), Ora-Plus (OP, B2), corn oil (CO, B3), or vehicle (Ora-Plus) alone (B4). Graph: mean ± range tumor volume (% baseline). **B.** Tumors harvested 4 hr after final OSI-906 dosing were stained for Ki67 and activated caspase 3. Scale bar 50 μm. **C.** Quantification of Ki67 (white bars) and activated caspase 3 (black bars). Ki67 counts were lower in group B4 (TMZ alone; *p < 0.05, **p < 0.01). Activated caspase 3 was detected only in tumors treated with TMZ followed by OSI-906. **D.** OSI-906 levels in plasma and tumors. Dotted line: steady state OSI-906 levels achieved at recommended Phase 2 dose [18]. OSI-906 levels in groups A (control) and B4 (TMZ alone) were below limits of quantitation: ~20 ng/ml (0.05 μM) for plasma, 200 ng/g (0.5 μM) for tumor. **E.** Mice bearing A375M xenografts were treated with Ora-Plus (control), 50 mg/kg TMZ (T), 50 mg/kg OSI-906 (O) or combination treatment, scheduling OSI-906 to avoid administration immediately before/with TMZ. Graph: mean ± SEM tumor volumes, *n* = 6. **F.** Graph: linear regression analysis of slopes of tumor growth, showing individual data points and mean ± SEM slope as % control. There were no differences in growth rates of control, TMZ or OSI-906 –treated tumors, and significant reduction in the combination group compared with each of the other groups (*p < 0.05, **p < 0.01).

To explore the therapeutic potential of these data, we tested whether OSI-906 is tolerable with TMZ *in vivo*, employing the sequential (TMZ → OSI-906) schedule identified as optimal *in vitro* (Figure 3D), and using A375M, known to be tumorigenic *in vivo* [27]. To mimic clinical treatment, TMZ was dosed on days 1–5 in Ora-Plus, as previously tested in glioblastoma [28]. After 5 days, TMZ-treated mice were divided into groups for IGF-1Ri dosing (Supplementary Figure S5A). OSI-906 is insoluble in aqueous solution, and has been previously dosed in tartaric acid [29]. In case this acid solvent could exacerbate toxicity, we compared OSI-906 administration in tartaric acid, Ora-Plus or corn oil (Figure 5A). TMZ and OSI-906 were tolerable when administered sequentially with weight loss generally <10%, with the exception of OSI-906 in tartaric acid (~12.5%), although OSI-906-treated groups were not significantly different (Supplementary Figure S5A–S5B). TMZ induced a trend to tumor growth delay (Figure 5A, Supplementary Figure S5C), but this experiment was not powered to detect differences in tumor volume. Four hours after final OSI-906 dosing, blood and tumor were collected for immunohistochemical and pharmacokinetic analysis. Ki67 positivity was detectable in all tumors, with evidence of reduction in the TMZ-alone group (Figure 5B, 5C), paralleling the reduced S-phase fraction in cell cycle analysis (Figure 4C). Activated caspase 3 was clearly detectable in tumors treated with TMZ followed by OSI-906, but not in vehicle or TMZ alone groups (Figure 5B–5C). OSI-906 dosing achieved drug levels in tumor of 3700 – 5700 ng/ml (8.7 – 13.6 μM), and plasma of ~4000 – 5600 ng/ml (9.3 – 13.3 μM; Figure 5D), corresponding to plasma levels achieved clinically [18, 19]. Ora-Plus was chosen to administer both TMZ and OSI-906, to assess effects on tumor growth.

Mice bearing A375M xenografts were treated with vehicle (Ora-Plus) alone, TMZ, OSI-906, or both drugs, using clinically relevant schedules, dosing TMZ on days 1–5 and OSI-906 on three days per week [1, 18]. This intermittent schedule allowed us to adopt the drug-sequencing regimen (TMZ → OSI-906) that was more effective *in vitro* (Figure 3D). After a second cycle of TMZ, OSI-906 dosing continued on a D1–3 q7 day cycle (Figure 5E). Both TMZ and OSI-906 induced weight loss <10%, which in the case of OSI-906 was rapid onset, unaccompanied by distress, and recovering rapidly on days off-treatment (Supplementary Figure S5D). Effects on tumor growth are shown in Figure 5E. Untreated control tumors grew rapidly, achieving a volume of 1000 mm^3^ in 29 ± 2 days from the start of treatment. TMZ or OSI-906 caused minor growth delay, taking 36 ± 6 and 39 ± 5 days respectively to reach 1000 mm^3^, not significantly different from controls (Supplementary Figure S5E). Combination treatment induced significant growth delay, tumors taking 79 ± 4 days to attain 1000 mm^3^ (*p* < 0.001 compared with the other groups, Supplementary Figure S5E). Growth delay was also evident when evaluating the gradient of tumor growth (Figure 5F), which was reduced by TMZ or OSI-906 by 13% and 25% respectively compared with controls. If additive, combination treatment would induce 38% reduction; the observed reduction was 78%, suggesting supra-additivity. Furthermore, survival was prolonged by the combination treatment compared with the other three groups (Supplementary Figure S5F). These results suggest that OSI-906 can be administered with TMZ to xenograft-bearing mice, and effects on melanoma growth are supra-additive compared with either agent alone.

## DISCUSSION

Our results indicate that human melanoma cell lines show variable sensitivity to IGF-1Ri, consistent with data from large drug screens in which A375 and SKmel2 were more sensitive to OSI-906 and A2058, CHL1 and SKmel28 more resistant [30]. Here, some cell lines showed potent inhibition of proliferation and cell survival at clinically-achievable OSI-906 concentrations. Indeed, the Phase 1 study of continuous oral dosing of OSI-906 reported an objective partial response, converted by surgery into a highly durable complete response, in one of 3 melanoma patients [19]. In the current study, responses to OSI-906 were unrelated to levels of total or activated IGF-1R, consistent with clinical findings that IGF-1R expression does not predict sensitivity to IGF-1R inhibition [31]. The most sensitive cell lines (501mel, A375, A375M; Table 1) harbor V600E BRAF; supporting activity in the context of activating RAS-RAF mutations, a study of synthetic lethal interactions identified IGF-1R as a key driver of AKT phosphorylation in KRAS mutant NSCLC [32]. In addition, we noted that in the melanoma cell lines and an isogenic NSCLC model, loss of WT p53 correlated with resistance to IGF-1R inhibition. The association of WT p53 with sensitivity to IGF-1R inhibition was also reported using the non-ATP-competitive IGF-1R inhibitor picropodophyllin [33], and may be relevant in selecting patients for treatment with IGF-1R inhibitory drugs.

The melanoma cell lines were relatively TMZ resistant, with GI_50_ and SF_50_ values above the clinical Cmax [1]. Our results indicate a correlation between intrinsic TMZ resistance and IGF-1R activation, and show that two different small molecule IGF-1R inhibitors sensitize human melanoma cells to TMZ. Both IGF-1Ri agents used here also inhibit INSR, and it would be informative to measure INSR expression in the melanoma cell line panel, to assess the contribution of this receptor to chemo-resistance. IGF-1R inhibitors have also been shown to enhance TMZ-sensitivity in neuroblastoma and medulloblastoma [34, 35]. These and other studies implicated IGF-1R as a mediator of chemo-resistance via regulation of AKT activation, cell cycle progression and cell survival [34–36]. In our study, IGF-1R-inhibited melanoma cells showed enhanced TMZ-induced apoptosis, relatively minor at 24–48 hr, more substantial 72 hr post-TMZ *in vitro*, and much more striking in melanoma xenografts after 5 days of TMZ and 3 days of OSI-906 (Figure 5B, 5C). These differences could reflect the greater dependence on IGF-1R for anchorage-independent 3D vs 2D growth [37], and/or the more prolonged time-course of the *in vivo* experiment.

In addition to regulating apoptosis, two additional mechanisms may be relevant to IGF-1R-mediated chemo-resistance. Firstly, cytotoxic drugs are reported to induce IGF-1R activation, which can be suppressed by IGF-1R inhibition [38]. Secondly, there is evidence from our group and others that a functional IGF-1R is required for DSB repair by HR, which is required for the repair of replication-associated DSBs induced by TMZ [12, 13, 39, 40]. However, combining IGF-1Ri with phase-specific chemotherapy raises concerns that sensitization to TMZ could be antagonized by delayed cell cycle progression in IGF-1R-inhibited cells. While we found no evidence of antagonism, we did observe more effective chemo-sensitization *in vitro* using sequential (TMZ → OSI-906) application (Figure 3D). We found no evidence that IGF-1R inhibition influenced removal of TMZ adducts (Supplementary Figure S2B–S2E), or delayed resolution of TMZ-induced DNA damage foci (Figure 4G), in contrast to the delayed repair of radiation-induced DSBs we reported recently [13]. There was, however, evidence that IGF-1R pre-inhibition caused accumulation of cells in G1, and reduced the subsequent yield of toxic TMZ-induced γH2AX foci (Figure 4C, 4G). Co-application of IGF-1R inhibitor showed the same phenotype, which is logical given the delay in induction of TMZ-induced DSBs. We also noted that the differences in γH2AX signal on western blot of A375M cells (Figure 4E, left) appeared to be more marked than the changes in γH2AX foci (Figure 4G), possibly implicating other processes such as apoptosis or replication stress that induce γH2AX [25].

Previous studies have recognized that delayed cell cycle progression induced by targeted agents including IGF-1Ri show potential to protect tumors against phase specific cytotoxic drugs [29, 41]. To our knowledge this protective effect has not until now been confirmed to be associated with reduced accumulation of toxic DNA damage. Unfortunately, this potential for antagonism has not been taken into account in the design of previous trials of IGF-1Ri with chemotherapy, and could have compromised outcomes in previous studies combining long half-life IGF-1R antibodies with phase-specific cytotoxic drugs [42–44]. Of potential relevance, we note that both A375M and CHL1 cells harbor mutant *CDKN2A*, which encodes mutant p16INK4a with reduced capacity to inhibit the cyclin D1/CDK4 complex that regulates the G1 to S transition [45]. Despite this, A375M cells responded to IGF-1Ri with accumulation in G1. While CHL1 cells were chemo-sensitized by IGF-1Ri, this effect did not vary with sequence, and nor did they show sequence-related changes in cell cycle distribution. It is possible that lack of schedule-dependence in this cell line could be due to the presence of mutant p53, while A375M cells harbour WT p53, and/or a reflection of the lesser sensitivity of CHL1 cells to IGF-1R inhibition. Although CHL1 cells did not show sequence-dependent differences in chemo-sensitivity, post-TMZ inhibition of IGF-1R was as effective as pre- or co- treatment. Therefore, the cytotoxic → IGF-1Ri sequence is preferred for future studies. Our data confirm that this sequential combination treatment regimen is tolerable *in vivo*, inducing supra-additive inhibition of melanoma xenograft growth, using doses and schedules of both drugs consistent with clinical use [1, 18].

In summary, our data support the concept that the IGF axis is an important mediator of therapy resistance. By exploiting interactions between the cell cycle, DNA damage response and apoptosis induction, IGF1R inhibition can be an effective route to chemo-sensitization. Our findings also highlight the importance of scheduling when combining IGF-1R inhibitory drugs and other targeted agents with cytotoxic drugs that induce replication-associated DNA damage.

## MATERIALS AND METHODS

### Cell lines and reagents

Melanoma cell lines CHL1, SKmel2, HMCB were from American Type Culture Collection, A375, A357M, 501mel and COLO858 from Professor Colin Goding (Ludwig Institute for Cancer Research Oxford), SKmel28 and A2058 from Cancer Research UK Cell Services, and SKmel23 from Professor Vincenzo Cerundolo (Weatherall Institute of Molecular Medicine Oxford). Subline B231 of H1299 p53 null non-small cell lung cancer (NSCLC) cells that express tetracycline-inducible wild-type p53 [20] was from Professor Xin Lu (Ludwig Institute for Cancer Research, Oxford). All cell lines were negative for mycoplasma (MycoAlert kit, Lonza Rockland Inc, Rockland US). AZ3801 and OSI-906 were provided by Elaine Kilgour (AstraZeneca) and Elizabeth Buck (OSI Pharmaceuticals/Astellas) respectively. TMZ was synthesized as described [46] and stored at +4°C protected from light. For *in vitro* use, 10 mM stock solutions of AZ3801, OSI-906, TMZ and O6BG (Sigma) in DMSO were aliquoted and stored at −20°C. Each agent was freshly diluted in culture medium to the correct final concentration. OSI-906 was freshly-prepared as a 10x stock in pre-warmed culture medium containing 10% FCS and 5% DMSO. Control cultures were treated with DMSO without drug.

### Western blotting, assays for viability, cell survival and apoptosis

CellTiter Glo (Promega) viability assays and clonogenic assays were performed as in reference [13], using cultures growing in full medium supplemented with 10% FCS. Western blotting was performed as described [13], using antibodies to: IGF-1R (#3027, Cell Signaling Technology, CST), phospho-Y1135/6 IGF-1R (#3024, CST), phospho-S473 AKT (#4051, CST), total AKT (#9272, CST), phospho-T202/Y204 ERK 1/2 (#4377, CST), total ERK 1/2 (#4695, CST), MGMT (#557045, BD Pharmingen), p53 (#9282, CST) PARP (#9542, CST) and actin (ab8224 or ab8227, Abcam). For each western blot shown, similar results were obtained in 1–2 further independent replicates. Viability and survival data were graphed and curve-fitted (GraphPad Prism v5) to interpolate GI_50_ and SF_50_ values (drug concentrations that suppress growth or survival to 50% of control values). Apoptosis was quantified by Apo-ONE^®^ Homogenous Caspase 3/7 Assay (Promega).

### Assays for MGMT, AAG, O^6^-meG and N7-meG

MGMT and AAG activity assays were based on cleavage of ^32^P labelled oligonucleotides containing single *O*^6^-meG or ethenoadenine residues as described [47, 48]. *O*^6^-meG levels in cellular DNA were determined by a modification of the MGMT activity assay [47] and levels of N7-meG adducts in cellular DNA were determined by an immunoslot blot method [49].

### Cell cycle analysis and immunofluorescence

Cell cycle analysis and detection of γH2AX foci were performed according to [13, 50]. Cells were pulsed with 10 μM 5-bromo-2′-deoxyuridine (BrdU; Sigma-Aldrich) for 30 min. Floating and adherent cells were collected, centrifuged at 250 g for 5 min and fixed in ice-cold 70% ethanol. After repeat centrifugation, cells were incubated in 2M HCl containing 0.1 mg/ml pepsin at 23°C for 20 min, washed with PBS and then with 2% FCS in PBS. Cells were resuspended in 2% FCS in PBS with αBrdU antibody (clone B44, BD Biosciences, 1:100) for 90 min at 23°C. After adding 2% FCS in PBS, the cells were pelleted again, resuspended in 2% FCS in PBS with Alexa Fluor 488 goat anti-mouse secondary antibody (Life Technologies, 1:200), and incubated for 60 min at 23°C in the dark. The cells were washed in PBS and resuspended in PBS containing 10 μg propidium iodide and 10 μl RNase (20 mg/ml, 70 U/ml; Invitrogen). After 15 min incubation at 23°C, samples were analyzed on a Becton Dickinson FACScan using FlowJo v 8.8.7 software.

For immunofluorescent detection of γH2AX foci, *c*ells were fixed with 4% paraformaldehyde with 0.1% Triton-X-100 in PBS for 10 min, permeabilized in 0.3% Triton-X-100 in PBS for 10 min and blocked in 3% BSA in PBS for 40 min. Cells were incubated overnight at 4°C with antibody to S139 γH2AX (#05–636, Upstate/Millipore), diluted 1:1000 in 3% BSA in PBS. After washing in PBS, bound antibody was detected with secondary antibody conjugated to Alexa Fluor 488 (InVitrogen; 1:1000 in 3% BSA) and incubated for one hour at 23°C in the dark. Cells were DAPI-stained, images were acquired on an IN Cell Analyzer 1000 (GE Healthcare) and analysed using IN Cell Analysis 3.5 software (GE Healthcare).

### Xenograft studies

*In vivo* work was carried out at Biomedical Services, John Radcliffe Hospital Oxford under a Home Office approved Project License. Xenografts were established by injecting 10^7^ A375M cells with matrigel (BD Biosciences) into the flanks of 6–7 week old female Balb/c immunodeficient (nu/nu) mice. Tumors were measured 2–3 times a week, volumes were calculated as π(length × width × height)/6, and mice were randomly allocated to treatment groups when tumor volumes reached 100–200 mm^3^. Treatments were prepared fresh each day, administered by gavage as a suspension of 2.5 mg/ml TMZ or OSI-906. Mice were weighed daily when on treatment to adjust dosing to 50 mg/kg, otherwise weekly. For initial tolerability and PK testing, mice were treated with 50 mg/kg TMZ in Ora-Plus (Fagron UK) on days 1–5, followed on days 6–8 by 50 mg/kg OSI-906 in Ora-Plus, 25 mM tartaric acid or corn oil. Controls were treated with Ora-Plus. Four hours after final OSI-906 dosing, the experiment was terminated, whole blood was obtained under anesthetic by cardiac puncture, and plasma was stored at −80°C. Tumors were halved for snap-freezing in liquid nitrogen, and formalin-fixation. OSI-906 levels in plasma and frozen tumor were analyzed by Andy Cooke and Mark Bittner (OSI Pharmaceuticals, Boulder CO). To test effects on tumorigenicity, mice were randomly allocated to four groups for dosing with Ora-Plus (control), TMZ on days 1–5 and 28–30, and/or OSI-906 on a three day per week schedule. Mice were sacrificed when tumors reached license limits or for loss of ≥20% baseline weight. Formalin-fixed, paraffin-embedded 4 μm xenograft sections were stained using antibodies to Ki67 (clone SP6, #VP-RM04, Vector Laboratories, 1:400 dilution) and active Caspase 3 (#AF835, R&D Systems, 1:1500). Ki67 positive nuclei were counted in 8 high-powered fields (0.25 mm^2^) for each section, and caspase 3 by the intensity (0–3 scale) and proportion (0–4) of positive staining, to generate Intensity × Percentage scores (IPS; 0–12).

### Statistical analysis

Data were analyzed using GraphPad Prism v5, using *t*-tests to compare 2 groups, one-way ANOVA for multiple groups, Spearman correlation for association between variables, and linear regression to compare xenograft growth rates.

## SUPPLEMENTARY FIGURE


