# Supplementary material for: IGF-1R inhibition induces schedule-dependent sensitization of human melanoma to temozolomide
Source: Oncotarget. 2015 Oct 15;6(37):39877–90. doi: 10.18632/oncotarget.5631 (PMC4741867; doi:10.18632/oncotarget.5631)
Supplement: Supplementary file 1 [file oncotarget-06-39877-s001.pdf]

SUPPLEMENTARY FIGURE

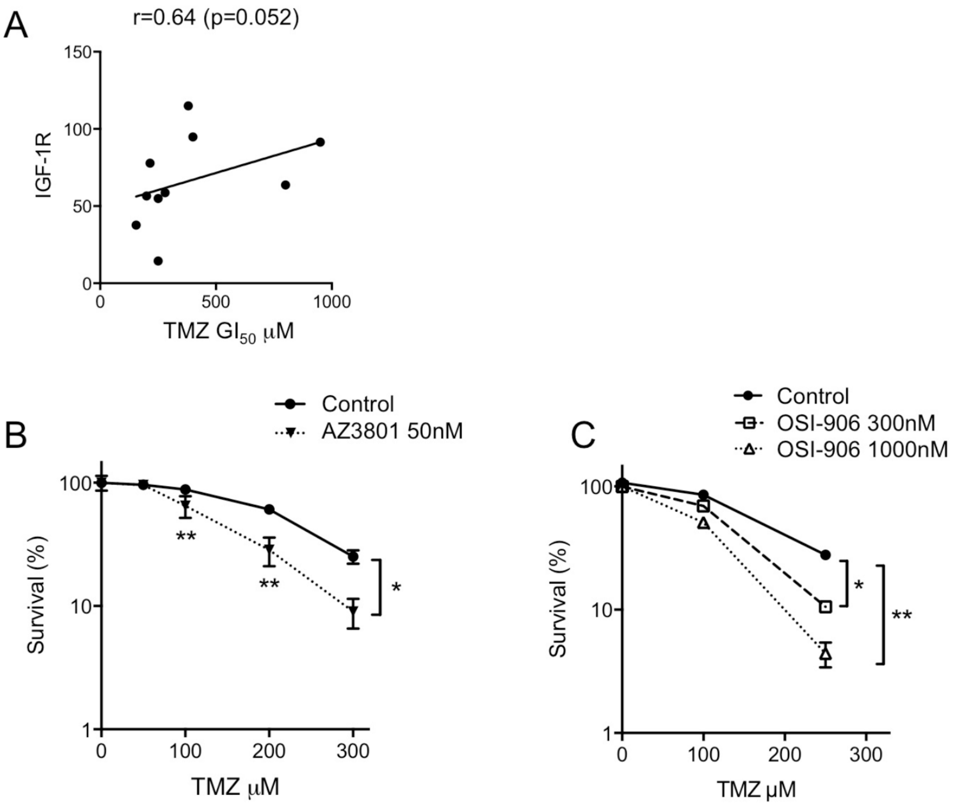

Supplementary Figure S1: Association of IGF-1R with chemo-resistance in melanoma. A. Graph shows trend to correlation between TMZ GI_{50} values and total IGF-1R protein, quantified from western blot shown in Figure 1A and one independent replicate, and corrected for actin loading. **B.** A375M cells were treated with AZ3801, and **C.** CHL1 cells with OSI-906, and 8–10 days later surviving colonies were counted and expressed as % survival in solvent-treated controls. Graphs show pooled data from 4 assays for A375M, 3 for CHL1, each with triplicate data points, expressed as mean ± SEM cell survival. A375M cells were sensitized by AZ3801 to TMZ (*$p < 0.05$, **$p < 0.01$ by two-tailed *t*-test), and CHL1 cells by OSI-906 (*$p < 0.05$, **$p < 0.01$ by ANOVA), similar to effects shown in Figure 2B, 2C.

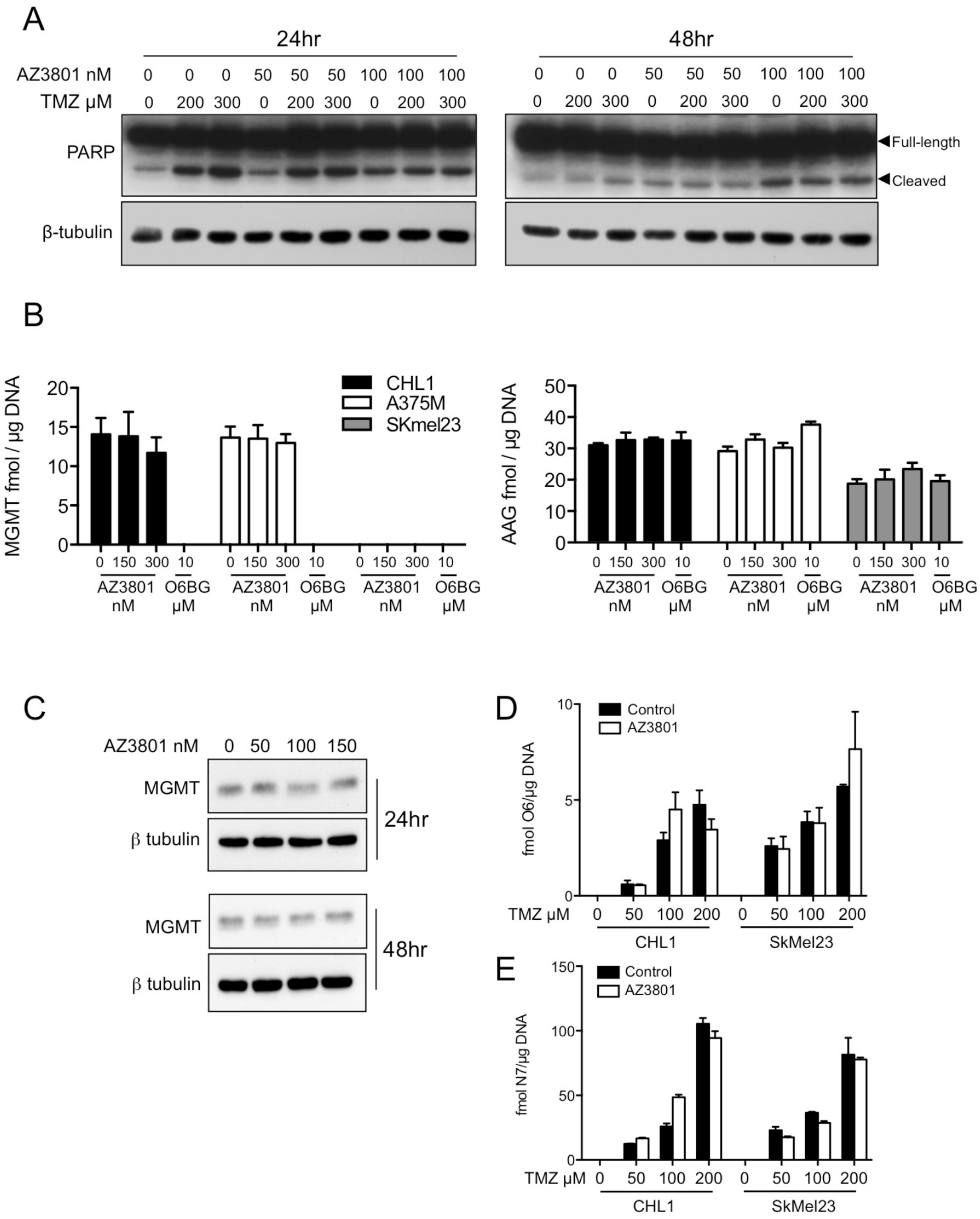

Supplementary Figure S2: IGF-1R inhibition induces apoptosis but does not influence MGMT expression or adducts formed in response to TMZ. **A.** CHL1 cells were treated with solvent, AZ3801 and/or TMZ and after 24 or 48 hr were lysed and western blotted for PARP cleavage. **B.** CHL1, A375M and SKmel23 cells were treated for 24 hr with solvent or AZ3801, or for 2 hr with 10 μM O6BG as a positive control for MGMT inhibition. Graphs show results of triplicate independent assays for MGMT or AAG activity expressed as mean ± SEM for: left, MGMT; right, AAG. **C.** CHL1 cells were treated with AZ3801 for 24 or 48 hr and analysed by western blot for MGMT. **D, E.** MGMT-proficient CHL1 and MGMT-deficient SKmel23 cells were treated with 50 nM AZ3801 for 4 hr prior to application of TMZ. After 2 hr, cells were harvested and assayed for D) O^6-meG and E) N7-meG adducts. The results are expressed as fmol adducts per μg DNA, mean ± SEM from three independent assays. IGF-1R inhibition did not significantly alter adduct yield.

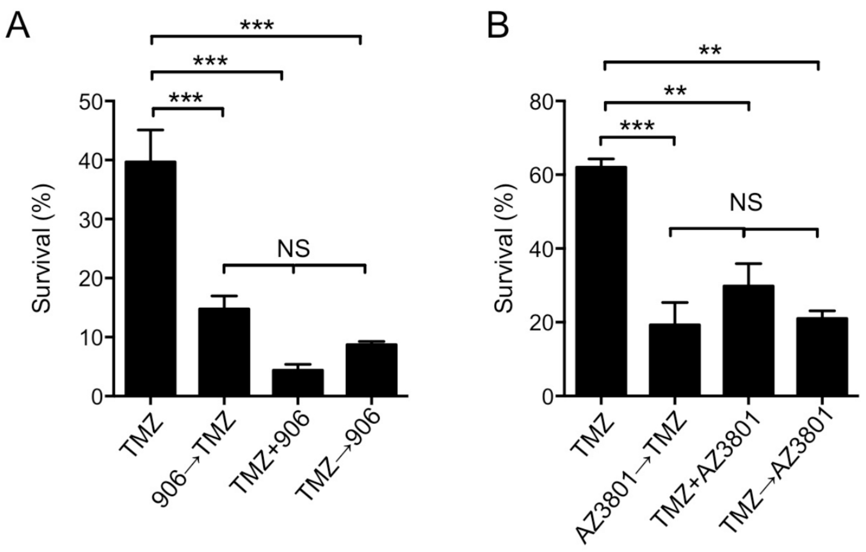

Supplementary Figure S3: IGF-1R inhibition induces sequence-independent chemo-sensitization of CHL1 melanoma cells. A. CHL1 cells were treated with solvent, 300 nM OSI-906 or 300 µM TMZ alone or with 300 nM OSI-906 applied 24 hr before, simultaneously or 24 hr after TMZ. Clonogenic survival was expressed as % survival in solvent controls for TMZ alone, and as % OSI-906 alone for TMZ plus OSI-906 combinations. All combinations of OSI-906 sensitized cells to TMZ (***$p < 0.001$) but there was no difference in relative cell survival with sequence of OSI-906. **B.** The experiment in A) was repeated, replacing OSI-906 with 50 nM AZ3801. As in Figure 2B, AZ3801 sensitized to TMZ (**$p < 0.01$, ***$p < 0.001$), but there was no difference between the different schedules of AZ3801.

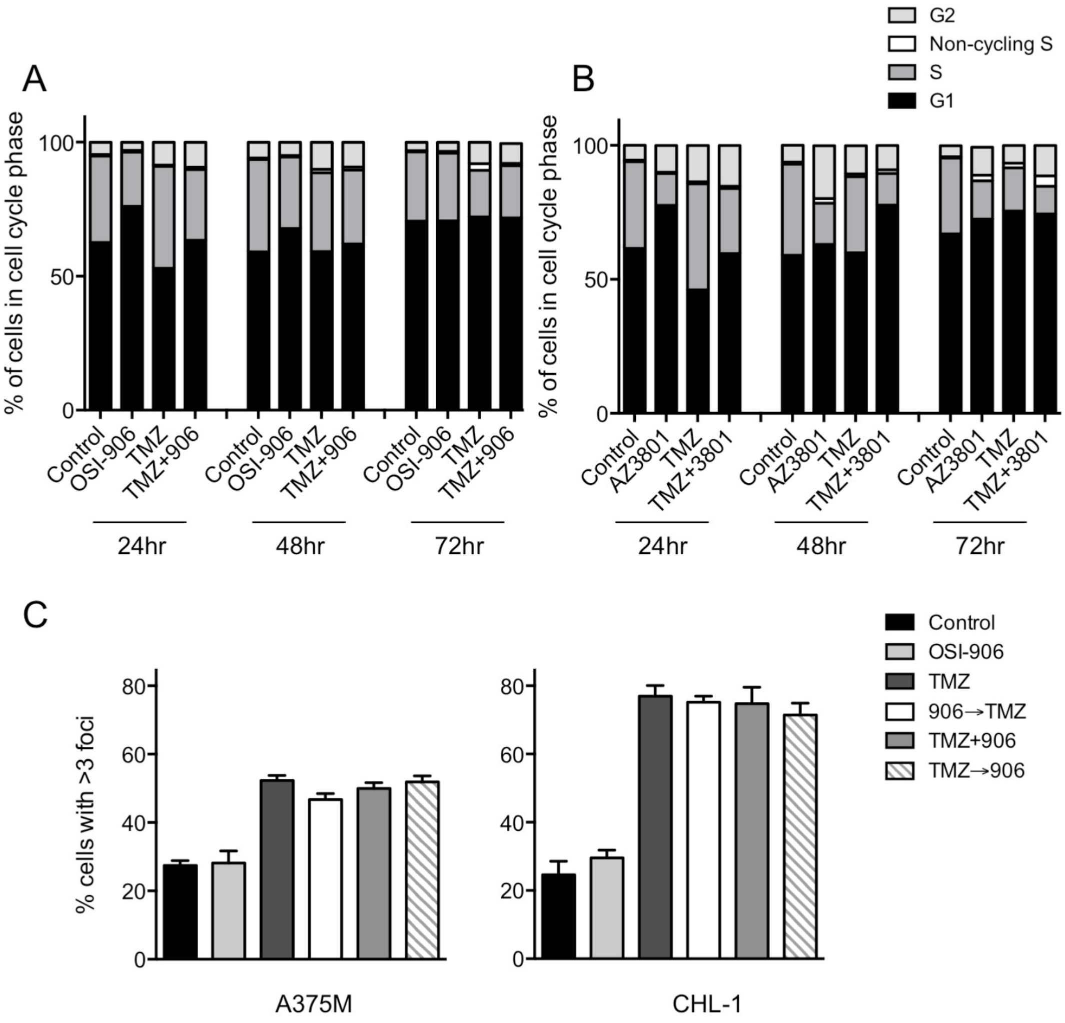

Supplementary Figure S4: Effects of IGF-1R inhibition on TMZ-induced changes in cell cycle distribution and γH2AX focus formation. A. A375M cells were treated with solvent, 300 nM OSI-906, 300 µM TMZ or the combination applied simultaneously, and harvested for cell cycle analysis after 24, 48 or 72 hr. **B.** A375M cells were treated, harvested and analysed as A), using 100 nM AZ3801 instead of OSI-906. **C.** Cells were treated with solvent, 300 nM OSI-906 alone, 300 µM TMZ alone or with 300 nM OSI-906 applied 24 hr before, simultaneously or 24 hr post-TMZ. After 72 hr, γH2AX foci were analyzed as in Legend to Figure 4G. Graphs show mean ± SEM proportion of cells with > 3 foci under each treatment condition. TMZ induced an increase in γH2AX foci in both A375M and CHL1 cells, but there were no significant differences with the addition of OSI-906.

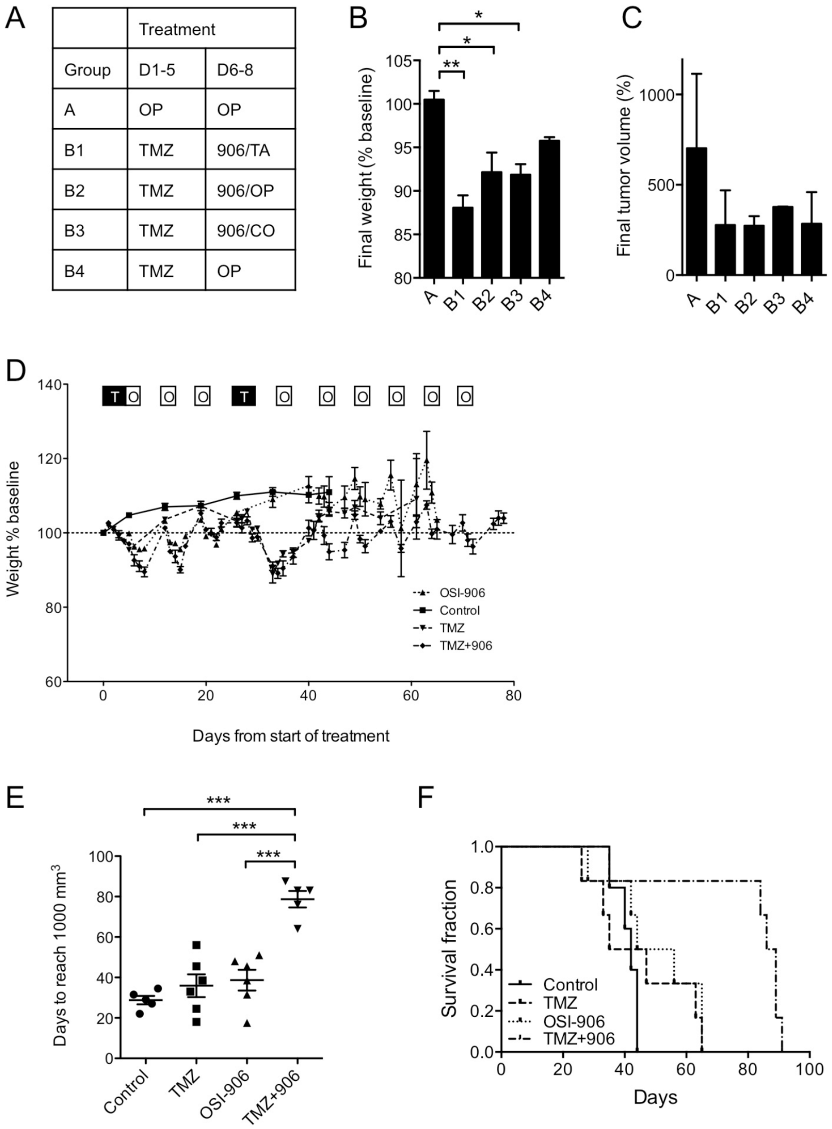

Supplementary Figure S5: The combination of OSI-906 with TMZ is tolerable and induces significant tumor growth delay. A. Mice bearing A375M xenografts were treated with Ora-Plus (Group A, 2 mice) or 50 mg/kg TMZ in Ora-Plus by gavage days 1–5 (Group B, 8 mice). On day 6, group B mice were randomly divided into 4 groups of 2 for gavage on days 6–8 with 50 mg/kg OSI-906 in 25 mM tartaric acid (TA, B1), Ora-Plus (OP, B2), corn oil (CO, B3) or vehicle (Ora-Plus) alone (B4). On day 8, final mouse weights **B.** and tumor volumes **C.** were recorded, graphed as mean ± range, and mice were culled 4 hr after final OSI-906 dosing. Compared with untreated controls (group A) there was significant weight loss in OSI-906-treated mice (*$p < 0.05$, **$p < 0.01$) that was slightly greater in group B1 treated with OSI-906 in tartaric acid (loss of 12 ± 1.5% baseline weight) compared with the other OSI-906 treatment groups (weight loss < 10%) although differences between OSI-906-treated groups (B1–3) were not significant, nor were final weights in these groups significantly different from the group treated with TMZ alone (B4). This experiment was not powered to detect differences in xenograft growth, but there was a trend to tumor growth delay in TMZ-treated mice compared with control group A. **D.** Mice bearing A375M xenografts were treated with 50 mg/kg TMZ (T) and/or 50 mg/kg OSI-906 (O) as Figure 5E, weights were measured every 1–3 days and expressed as % baseline (pre-treatment) weight. Mice were not dosed if weight loss was >10% baseline weight, leading to omission of single doses on 2, 2 and 4 occasions in the TMZ, OSI-906 and combination (TMZ plus OSI-906) groups respectively. One animal in each of the TMZ and combination groups experienced 20% weight loss and was culled on welfare grounds. Otherwise, weight loss was generally < 10%, and there was no other apparent toxicity. **E.** Tumor volume data from Figure 5E were used to determine time to reach tumor volume of 1000 mm^3. Graph shows individual data points, mean and SEM. There was a significant delay in time to reach 1000 mm^3 in the combined (TMZ+906) group compared with each of the other groups (***$p < 0.001$ by one way ANOVA). **F.** Survival from start of treatment. Survival differences were calculated by log-rank Cox-Mantel test with Bonferroni-correction for multiple comparisons. There were no differences between control, TMZ and OSI-906 -treated groups, while survival in the combination group was significantly longer than control ($p = 0.0082$), TMZ-treated ($p = 0.0026$) and OSI-906-treated ($p = 0.0059$) groups.
